# Separation of americium from highly active raffinates by an innovative variant of the AmSel process based on the ionic liquid Aliquat-336 nitrate

**DOI:** 10.1039/d3ra06064k

**Published:** 2023-12-12

**Authors:** Filip Kolesar, Karen Van Hecke, Péter Zsabka, Ken Verguts, Koen Binnemans, Thomas Cardinaels

**Affiliations:** a Belgian Nuclear Research Center (SCK CEN), Institute for Nuclear Materials Science Boeretang 200 2400 Mol Belgium karen.van.hecke@sckcen.be; b KU Leuven, Department of Chemistry Celestijnenlaan 200F, P.O. Box 2404, Leuven 3001 Belgium

## Abstract

A new variant of the AmSel (Americium Selective Separation) system for the separation of Am(iii) from a PUREX raffinate was tested in which the aliphatic diluent was replaced by the ionic liquid Aliquat-336 nitrate. For this ionic liquid variant, the kinetics, and the influence of both the HNO_3_ concentration and the ligand concentration on the stripping were evaluated. In addition, both the original AmSel system, and the ionic liquid variant were demonstrated on a simulated highly active raffinate. The introduction of Aliquat-336 nitrate results in an improved separation between Am(iii) and the fission products, in particularly for the light lanthanides and strontium. The Am/Cm separation factors of the ionic liquid variant were found to remain similar to the original AmSel process. Despite the improved separation, slower stripping kinetics were observed and extraction of the SO_3_-Ph-BTBP complexant to the Aliquat 336 nitrate phase occurred at low HNO_3_ concentrations during the stripping step. However, adequate mitigation actions to counteract these issues were found and applied.

## Abstract 2

A new variant of the AmSel (Americium Selective Separation) system for the separation of Am(iii) from a PUREX raffinate was tested in which the aliphatic diluent was replaced by the ionic liquid Aliquat-336 nitrate.

## Graphical abstract

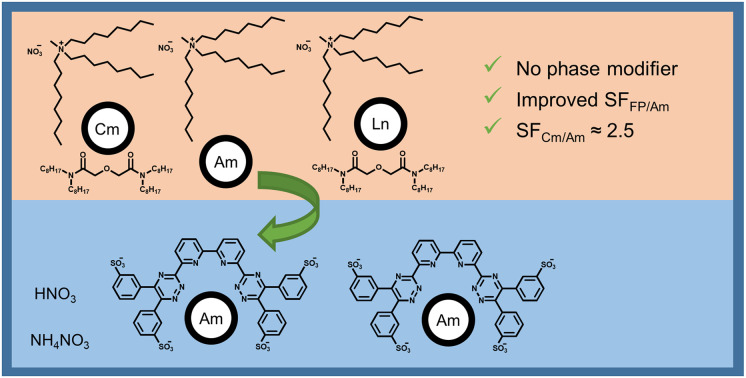

## Introduction

**Fig. 1:**
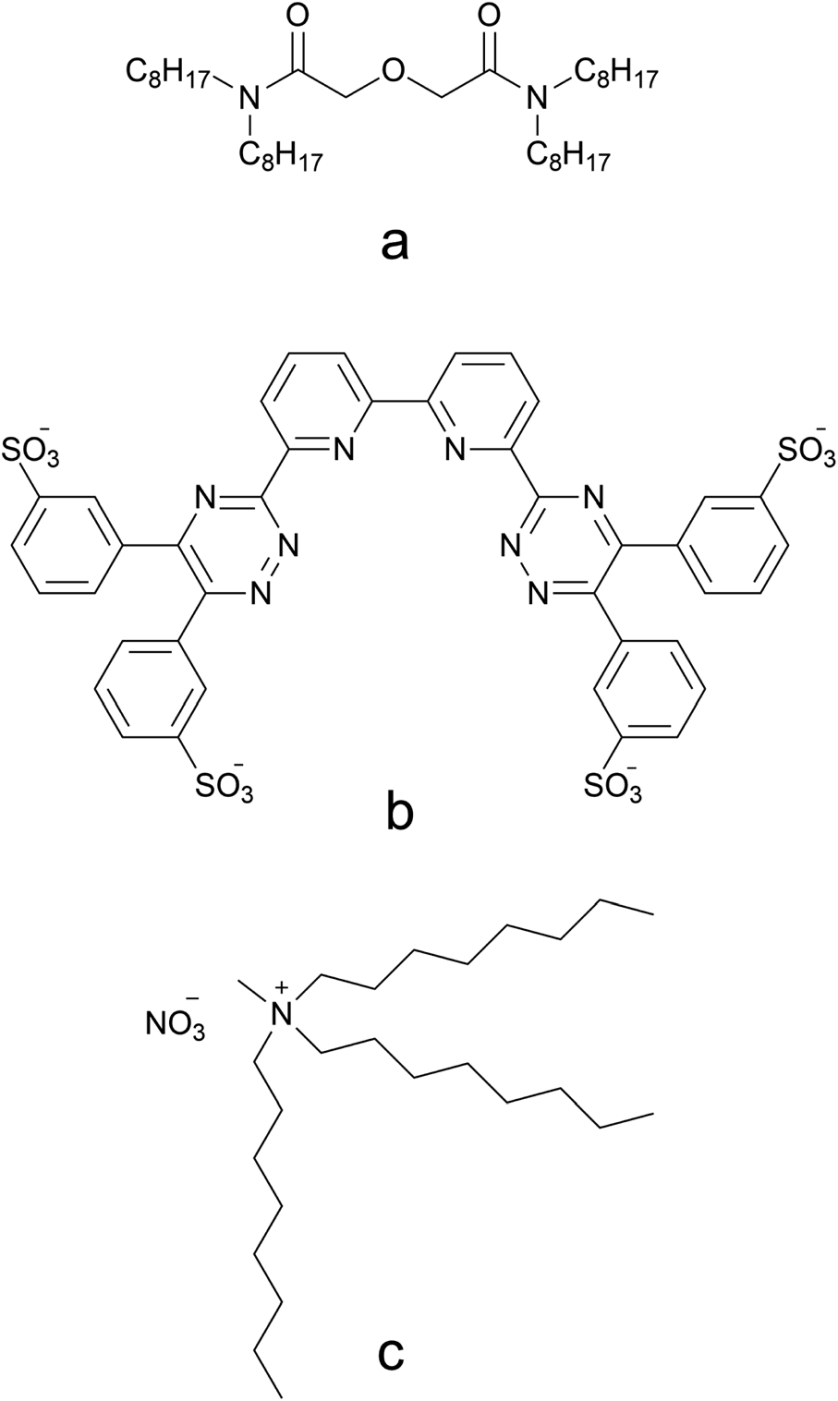
Chemical structures of TODGA (a), SO_3_-Ph-BTBP (b), and *N*-methyl-*N*,*N*,*N*-trioctyl ammonium nitrate, the main component of Aliquat-336 nitrate (c).

With global energy consumption expected to rise nearly 50% by 2050, and an ever growing awareness of the effects of burning fossil fuels on the environment, nuclear energy has regained interest as a potential source of electricity.^1,2^ A persistent problem however is the spent nuclear fuel that remains radiotoxic over long periods of time. A partitioning and transmutation strategy has been theorized as a solution to the growing stock of spent nuclear fuel. In theory, this strategy could significantly reduce the time within which the residual radiotoxicity of the highly active spent nuclear fuel is reduced to the level of uranium ore (from 250 000 years to less than 300 years).^3^ However, such a reduction of radiotoxicity is only feasible, if in addition to the construction of fast reactors, a full-scale multi-recycling of both major actinides and minor actinides is implemented. Uranium and plutonium are already being partitioned on an industrial scale using the well-developed PUREX process at La Hague (France), Mayak (Russia), and until recently at Sellafield (United Kingdom).^4^ Neptunium could theoretically also be partitioned with a few adaptions to the PUREX process.^5^ After the removal of U, Pu and Np, minor actinides, mainly americium and curium, are responsible for the majority of the long-term radiotoxicity and decay heat.^3^ However, no industrial process for the treatment of high level liquid waste resulting from the PUREX process has yet been implemented. A number of strategies are being pursued, amongst which are solvent extraction methods without change of the oxidation state of americium, solvent extraction methods with selective oxidation of americium, and column chromatography.^6^ So far much of research into oxidation methods has focused on the insoluble NaBiO_3_ as an oxidizing agent,^7^ although recent studies have shown copper(iii) periodate to be a promising, soluble, alternative.^8,9^ This selective oxidation is then followed by a selective extraction of the oxidized Am by an extractant such as TBP.^10^ The solvent extraction systems for selective americium separation find their origin in actinide–lanthanide separation systems whose development started in the 1960s. Amongst the first processes developed for this actinide–lanthanide separation is the TALSPEAK (Trivalent Actinide Lanthanide Separation by Phosphorus-Reagent Extraction) process. This system combines an organophosphoric acid, such as di(2-ethylhexyl)phosphoric acid (HDEHP), and carboxylic acids to achieve the separation.^11^ Combination of this process with the TRUEX (Trans Uranium Extraction) process has led to the development of TRUESPEAK. The lowest obtained separation factor in this system is that between americium and samarium, and has a value of around 12.^12^ Several solvent extraction systems have also been developed within European framework programmes (NEWPART,^13^ EUROPART,^14^ GENIORS,^15^*etc.*), focusing primarily on the CHON principle, whereby solvents and extractants only consist of carbon, hydrogen, oxygen, and nitrogen in order to avoid solid secondary waste. Within these programmes, DIAMEX (Diamide Extraction) is one of the promising systems, which originally employed diamide extractants capable of co-extracting actinides and lanthanides together.^16,17^ An important improvement of the DIAMEX process was the replacement of diamides by diglycolamides such as TODGA (*N*,*N*,*N′*,*N′*-tetraoctyl diglycolamide, see Fig. 1a) which show higher affinities for both the actinide and lanthanide ions, and are more resistant towards radiolysis.^18–20^ The SANEX (Selective Actinide Extraction) process was developed as a follow up process to the DIAMEX process, to separate the actinides from the lanthanides.^21,22^ In SANEX, a class of ligands based on bis-triazinyl bipyridines (BTBPs) is used, which selectively separates the minor actinides from the lanthanides. The optimized extractant within SANEX is often referred to as CyMe_4_-BTBP.^23^ Combination of these two processes has led to the development of 1-cycle SANEX^24^ and ALSEP^25^ (Actinide–Lanthanide Separation Process) which achieve the selective actinide separation in a single cycle. In another variant, the i-SANEX (innovative SANEX) process, a hydrophilic bis-triazinyl pyridine (BTP) molecule is used to selectively strip the actinides from a loaded organic phase instead of selectively extracting the actinides from an aqueous phase.^26^ However, one of the issues with all of these processes is the inability to separate curium from americium. The relatively short-lived isotopes of curium show high neutron emissions, which requires thick shielding and would therefore complicate the reprocessing, manufacturing, and handling of minor actinide containing fuel. Furthermore, these neutron emissions are cumbersome for reactor operation. The long-term contribution of curium towards radiotoxicity is however small and it is considered much more practical to store curium and allow for its decay (^244^Cm has a half-life of 18 years).^27^ Despite the benefits of such a strategy, the separation of americium from curium is a challenge as both elements usually occur as trivalent cations with similar ionic radii (0.98 Å for Am^3+^ and 0.97 Å for Cm^3+^).^28–30^ This led to the development of the EXAm process, which evolved from the DIAMEX-SANEX process.^31–34^ The Am/Cm separation is achieved by selective extraction of Am with *N*,*N*′-dimethyl,*N*,*N*′-dioctylhexylethoxymalonamide (DMDOHEMA) and HDEHP in a hydrogenated tetrapropylene (HTP) diluent. In this system, curium is kept in the aqueous phase as a complex with *N*,*N*,*N*′,*N*′-tetraethyldiglycolamide (TEDGA). Together with the minor actinides, also some of the lighter lanthanides as well as certain fission products are co-extracted into the organic phase. This necessitates an intermediate scrubbing stage to remove Mo and Ru, after which Am can be selectively stripped with a combination of *N*-(2-hydroxyethyl)ethylenediamine-*N*,*N*′,*N*′-triacetic acid (HEDTA) and diethylenetriaminepentaacetic acid (DTPA).^34^ Selective Am stripping has also been achieved with the BTP family of complexants. The hydrophilic 3,3′,3′′,3′′′-[3-(1,10-phenanthroline-2,9-diyl)-1,2,4-triazine-5,5,6,6-tetrayl] tetrabenzenesulfonic acid (SO_3_-Ph-BTPhen)^35,36^ and 3,3′,3′′,3′′′-([2,2′-bipyridine]-6,6′-diylbis(1,2,4-triazine-3,5,6-triyl)) tetrabenzenesulfonate (SO_3_-Ph-BTBP)^37,38^ – see Fig. 1b – have shown selectivity towards the stripping of Am. In the AmSel system (Americium Selective Extraction), the lanthanides and minor actinides are first extracted into an organic phase using TODGA as extractant, after which Am is selectively stripped using either SO_3_-Ph-BTPhen or SO_3_-Ph-BTBP as complexant. The AmSel system relies on a hard donor ligand in the organic phase (TODGA) and a soft donor ligand in the aqueous phase (SO_3_-Ph-BTPhen or SO_3_-Ph-BTBP) to achieve separation. The An(iii) form slightly stronger complexes with soft donor atoms than the Ln(iii) due to the stronger covalent interactions of the soft donor ligands with the actinide's 5f orbitals than with the lanthanide's 4f orbitals.^39,40^ The small difference in ionic radius between Am^3+^ and Cm^3+^ allows for their separation under specific conditions.

Relatively unexplored in the field of spent nuclear fuel partitioning are ionic liquids (ILs), which are salts with a melting temperature below 100 °C. Ionic liquids can be employed both as diluent or as extractant. An example of the latter are the task-specific ionic liquids developed by Ouadi *et al.* for extraction of trivalent americium by attaching a 2-hydroxybenzylamine moiety to a 1-methylimidazolium core with counterions being either hexafluorophosphate, [PF_6_]^−^, or bis(trifluoromethylsulfonyl)imide, [Tf_2_N]^−^.^41^ Ionic liquids offer several advantages over common aliphatic and aromatic hydrocarbons and have the potential to greatly improve partitioning of spent nuclear fuel. One of these advantages is that ILs are in general more resistant to radiation. This was evaluated in a recent study which tested the radiolytic stability of TODGA and CyMe_4_-BTPhen in the ionic liquid *N*-methyl-*N*,*N*,*N*-trioctyl ammonium nitrate [N_1888_][NO_3_] – see Fig. 1c – TODGA showed higher resistance towards radiolysis when compared to lipophilic DGAs, including TODGA, in *n*-dodecane after exposure to similar radiation doses.^20,42,43^ The main disadvantages of ionic liquids are their higher viscosity and slower phase separation compared to aliphatic and aromatic diluents.^44^ One very promising ionic liquid for minor actinide partitioning is Aliquat-336 nitrate ([A336][NO_3_]), of which trioctylmethlyammonium nitrate, [N_1888_][NO_3_], is the main component. Its advantages are a higher flash point than the commonly used aliphatic diluents (132 °C for Aliquat-336 and 84 °C for *n*-dodecane), high loading without third-phase formation, and being compliant with the CHON principle. A solution consisting of TODGA in the quaternary ammonium salt Aliquat-336 nitrate was shown to be able to extract Am(iii), Cm(iii) and Ln(iii) ions from a simulated Highly Active Raffinate (HAR).^45^ This extraction was proven to be reversible, with no third-phase formation nor precipitation observed during either extraction or stripping. Selective Am/Cm and Am/Ln separation has been realized with a system consisting of CyMe_4_-BTPhen in Aliquat-336 nitrate ionic liquid which has displayed promising results, with Am/Cm separation factors of up to 17 and Am/Ln separation factors higher than 75.^46^

In this paper, a new extraction system is presented that expands upon the AmSel system developed by Wagner *et al.* by introducing Aliquat-336 nitrate as the organic diluent.^38^ This work also further expands upon previous research on extraction of lanthanides and actinides with a combination of TODGA and Aliquat-336 nitrate.^45^ The selective separation of americium was realized through the inverse selectivity of TODGA for the lanthanides and curium, and SO_3_-Ph-BTBP for americium. The kinetics of the stripping and the influence of acid and ligand concentration were studied and compared to the aliphatic AmSel system. Furthermore, both the aliphatic and ionic liquid AmSel systems were demonstrated on a simulated highly active raffinate.

## Experimental

### Chemicals

HNO_3_ solutions were prepared from 69% trace metal grade HNO_3_, acquired from Fischer Scientific. Y(NO_3_)_3_·6H_2_O (purity 99.9%), Ce(NO_3_)_3_·6H_2_O (purity 99.9%), Pr(NO_3_)_3_·6H_2_O (purity 99.9%), Nd(NO_3_)_3_·6H_2_O (purity 99.9%), and Sm(NO_3_)_3_·6H_2_O (purity 99.9%) were obtained from Strem Chemicals (Kehl, Germany). La(NO_3_)_3_·6H_2_O (purity 99.0%) was procured from Fluka (Seelze, Germany). Eu(NO_3_)_3_·6H_2_O (purity 99.99%) and Gd(NO_3_)_3_·6H_2_O (purity 99.9%) were acquired from Alfa Aesar GmbH (Karlsruhe, Germany). Dy(NO_3_)_3_·6H_2_O (purity 99.9%), Yb(NO_3_)_3_·5H_2_O (purity 99.9%), ACS grade 1-octanol and ReagentPlus grade *n*-dodecane were obtained from Sigma-Aldrich (Steinheim, Germany). From the lanthanide nitrates a stock solution was prepared by dissolving weighed amounts of the Y, La, Ce, Pr, Nd, Sm, Eu, Gd, and Dy nitrate salts in 0.1 mol L^−1^ HNO_3_ solution to obtain a 10^−3^ mol L^−1^ concentration for each of the elements. This solution was then diluted with 3 mol L^−1^ HNO_3_ solution to obtain a concentration of 10^−5^ mol L^−1^ for each lanthanide (a total lanthanide concentration *ca.* 2000 times lower than in the simulated HAR). The exact HNO_3_ concentration was determined through titration with 0.05 mol L^−1^ NaOH in a Mettler Toledo Titration Excellence T5 autotitrator. TODGA and SO_3_-Ph-BTBP were acquired from Technocomm Limited (Edinburgh, UK). Aliquat^®^ 336 chloride TG was purchased from Alfa Aesar (Ward Hill, USA). Aliquat-336 chloride was converted to Aliquat-336 nitrate through a metathesis reaction by stirring equal volumes of [A336][Cl] and a 2.5 mol L^−1^ KNO_3_ solution, followed by phase separation.^47^ The metathesis reaction was repeated until no AgCl formation was observed upon addition of AgNO_3_. In general, seven repetitions of this metathesis reaction were needed. All solutions and dilutions were prepared with Milli-Q^®^ grade water. ^241^Am radiotracer solution in 1 mol L^−1^ HNO_3_ (radionuclide purity >99%) was available from the legacy stocks of SCK CEN. ^244^Cm tracer (radionuclide purity >99.9%) in 1 mol L^−1^ HNO_3_ solution and ^152^Eu tracer (radionuclide purity >99%) in 0.5 mol L^−1^ HNO_3_ solution were obtained from Eckert and Ziegler Nuclitec GmbH (Braunschweig, Germany). These radiotracers were combined into a mixed tracer stock solution consisting of 0.1 mol L^−1^ HNO_3_ solution with 300 kBq mL^−1^ of each radiotracer.

A simulated (non-radioactive) highly active raffinate (HAR) was prepared based on an in-house calculation of the composition of spent LWR UO_*x*_ fuel. This fuel is assumed to have an initial ^235^U enrichment of 4.2%, a burn-up of 50 GWd/t_HM_, and a cooling time of 10 years between the unloading and reprocessing of the spent fuel. This HAR simulates the composition of a PUREX raffinate that has a dissolution volume of 5000 L per metric tons of UO_*x*_ fuel. Cu(NO_3_)_2_·3H_2_O (purity 99.5%), Ni(NO_3_)_2_·6H_2_O (purity 99.9%), RbNO_3_ (purity 99%), Rh(NO_3_)_3_ (10% solution), Sr(NO_3_)_2_ (purity 99%), Zn(NO_3_)_2_·6H_2_O (purity 98%), and H_2_TeO_4_·2H_2_O (purity 99.5%) were acquired from Strem chemicals (Kehl, Germany). CsNO_3_ (purity 99.98%), Pd(NO_3_)_2_ (purity 99.8%), (NH_4_)_6_Mo_7_O_24_·4H_2_O (purity 99.8%), and Ru(NO)(NO_3_)_3_ (31.3% Ru) were obtained from Alfa Aesar GmbH (Karlsruhe, Germany). Ba(NO_3_)_2_ (purity 99%) and Cr(NO_3_)_3_·9H_2_O (purity 99%) were acquired from Fluka (Seelze, Germany). ZrO(NO_3_)_2_·*x*H_2_O (purity 99%), and ACS grade NaNO_3_ and Fe(NO_3_)_3_·9H_2_O were obtained from Sigma-Aldrich, (Steinheim, Germany). The solution was prepared by first dissolving ZrO(NO_3_)_2_ and Ba(NO_3_)_2_ in 50 mL concentrated (10 mol L^−1^) nitric acid through stirring and heating the solution for at least 3 days. Two other separate solutions were made: one in which all the lanthanide nitrates and Y(NO_3_)_3_ were dissolved in 50 mL of 0.1 mol L^−1^ HNO_3_ solution, and one where the remaining fission products apart from Fe(NO_3_)_3_ and NaNO_3_ were dissolved in 50 mL of 1 mol L^−1^ HNO_3_. Once all the metal nitrates were dissolved, the three solutions were combined in a 500 mL volumetric flask and Fe(NO_3_)_3_ and NaNO_3_ were added. The solution was then finally diluted with water and HNO_3_ until the desired volume and HNO_3_ concentration were reached. The HNO_3_ concentration was determined by titration to be 4.1 mol L^−1^. The exact composition of this simulated solution was determined by ICP-MS and is represented Table 1a. The uncertainty on the concentrations is 10%.

**Table 1:** ICP-MS analysis results of simulated HAR feed solution in 4 mol L^−1^ HNO_3_ (a). Extraction efficiency of extraction with TODGA in aliphatic organic phase (b). Stripping efficiency of back extraction from loaded aliphatic organic phase with SO_3_-Ph-BTBP (c). Extraction efficiency of extraction with TODGA in [A336][NO_3_] organic phase (d). Stripping efficiency of back extraction from loaded [A336][NO_3_] organic phase with SO_3_-Ph-BTBP (e)^a^

Element	a	b	c	d	e
	HAR starting composition (mg L^−1^)	Extraction efficiency aliphatic AmSel (%)	Stripping efficiency aliphatic AmSel (%)	Extraction efficiency ionic liquid AmSel (%)	Stripping efficiency ionic liquid AmSel (%)
Cr	67	<1	—	<1	—
Fe	172	<1	—	<1	—
Ni	41	<1	—	<1	—
Cu	19.5	<1	—	<1	—
Zn	23.9	<1	—	8.4	<1
Rb	103	<1	—	<1	—
Sr	208	56	>99	14	>99
Y	130	>99	<1	>99	<1
Zr	990	9.1	13	9.1	<1
Mo	920	18	>99	10	>99
Ru	760	26	7.4	45	3.9
Rh	118	<1	—	<1	—
Te	141	<1	—	1.4	4.1
Cs	690	<1	—	<1	—
Ba	500	<1	<1	10	<1
La	350	>99	22	97	3.8
Ce	660	>99	14	>99	1.8
Pr	320	>99	8.9	>99	<1
Nd	1160	>99	4.5	>99	<1
Sm	276	>99	<1	>99	<1
Eu	45	>99	<1	>99	<1
Gd	52	>99	<1	>99	<1
Dy	37.2	>99	<1	>99	<1

a Experimental conditions: org phase: 0.2 mol L^−1^ TODGA in 10 vol% 1-octanol in *n*-dodecane. Aq phase: Simulated HAR in 4 mol L^−1^ HNO_3_ + radiotracer spike. Shaking time = 1 h. *T* = 20 °C, A/O = 1 (b). Org phase: loaded 0.2 mol L^−1^ TODGA in 10 vol% 1-octanol in *n*-dodecane. Aq phase: 4 mmol L^−1^ SO_3_-Ph-BTBP in 0.3 mol L^−1^ HNO_3_. Shaking time = 1 h. *T* = 20 °C, A/O = 1 (c). Org phase: 0.2 mol L^−1^ TODGA in [A336][NO_3_]. Aq phase: Simulated HAR in 4 mol L^−1^ HNO_3_ + radiotracer spike. Shaking time = 1 h. *T* = 20 °C, A/O = 1 (d). Org phase: loaded 0.2 mol L^−1^ TODGA in [A336][NO_3_]. Aq phase: 50 mmol L^−1^ SO_3_-Ph-BTBP + 3 mol L^−1^ NH_4_NO_3_ in 0.5 mol L^−1^ HNO_3_. Shaking time = 90 min. *T* = 20 °C, A/O = 1 (e).

### Methods

All extraction experiments were performed twofold. An inactive experiment was performed to study the behavior of the stable lanthanides by means of ICP-MS and of the ligand by means of UV-VIS spectrometry. Radioactive experiments were performed to study the behavior of the radioactive tracers using gamma spectrometry (for ^241^Am and ^152^Eu) and alpha spectrometry (for ^241^Am and ^244^Cm). The separation between Am and Cm was followed through alpha spectrometry. ^152^Eu was added to represent the lanthanides, and separation between Eu and Am was determined through gamma spectrometry and compared to existing literature. The starting aqueous phase consisted of a mixed metal solution in a 3 mol L^−1^ HNO_3_ solution, with or without a mixed radiotracer spike. 10 μL of mixed radiotracer solution (3 kBq of each radiotracer) was added per 1000 μL of lanthanide starting solution or simulated HAR solution. The organic phases consisted of 0.2 mol L^−1^ TODGA in Aliquat-336 nitrate, *i.e.* the same TODGA concentration used in the aliphatic AmSel process.^38^

Extractions were performed by combining equal amounts (0.5 mL or 1 mL) of aqueous phase and organic phase in a glass 4 mL vial. The aqueous over organic phase ratio (A/O ratio) was therefore 1 in each experiment. Samples were then mechanically shaken using a TMS-200 Thermoshaker at 1900 rpm and at a temperature of 20 °C for 90 min to reach equilibrium. The temperature was controlled by an RC10 VWR digital chiller. After shaking, the vials were centrifuged for 4 min at 4500 rpm to achieve complete phase separation. The phases were then separated by pipetting them into new vials using Eppendorf Research Plus pipettes.

Prior to the stripping experiments, a loading step was performed on a larger volume of organic phase. This was done to create a uniform starting organic phase and to avoid variations in the starting conditions of stripping experiments. First the organic phases were pre-equilibrated twice by combining equal volumes of 0.2 mol L^−1^ TODGA in Aliquat-336 nitrate and 3 mol L^−1^ HNO_3_ solution in a 50 mL Falcon® tube and shaking for 15 min at room temperature on a vortex mixer. Then the organic phase was combined with an equal volume of 10^−5^ mol L^−1^ mixed lanthanide solution with a concentration of 3 mol L^−1^ HNO_3_, with or without radiotracer spike, and shaken for 30 min at room temperature on a vortex mixer. The phases were then separated by pipetting them into new vials, and the interface was centrifuged to achieve a full separation. The organic phase was then used in stripping experiments. Stripping solutions consisted of SO_3_-Ph-BTBP and NH_4_NO_3_ dissolved in HNO_3_. These were prepared by weighing the required amount of ligand on an analytical balance and dissolving this amount in a HNO_3_ solution with the required concentration. When very low concentrations of ligand were used, first a more concentrated stock solution was prepared by weighing SO_3_-Ph-BTBP on an analytical balance, and this solution was then diluted until the required concentration was obtained.

#### Influence of the NH_4_NO_3_ concentration

To study the influence of the NH_4_NO_3_ concentration, aqueous phases consisting of 20 mmol L^−1^ SO_3_-Ph-BTBP and 0.5, 1, 2, 3, 4, or 5 mol L^−1^ NH_4_NO_3_ in 0.5 mol L^−1^ HNO_3_ solution, were shaken with an organic phase consisting of bulk loaded 0.2 mol L^−1^ TODGA in Aliquat-336 nitrate. The samples were shaken for 90 min at 20 °C, centrifuged, and the phases separated and analyzed. Apart from the NH_4_NO_3_ concentration, all conditions were kept constant throughout the experiment.

#### Kinetics

To study the kinetics, identical samples were shaken at 1900 rpm for various periods of time, after which the phases were separated and analysed. The organic phase consisted of bulk loaded 0.2 mol L^−1^ TODGA in Aliquat-336 nitrate and the aqueous phase consisted of 20 mmol L^−1^ SO_3_-Ph-BTBP and 3 mol L^−1^ NH_4_NO_3_ in 0.5 mol L^−1^ HNO_3_. The phases were contacted with each other and shaken at 20 °C for 15, 30, 60, 90, 120 or 150 min.

#### Influence of the HNO_3_ concentration

To study the influence of the HNO_3_ concentration, aqueous phases consisting of 20 mmol L^−1^ SO_3_-Ph-BTBP and 3 mol L^−1^ NH_4_NO_3_ in 0.10, 0.25, 0.50, 0.75, 1.0, or 2.0 mol L^−1^ HNO_3_ were shaken with an organic phase consisting of bulk loaded 0.2 mol L^−1^ TODGA in Aliquat-336 nitrate. The samples were shaken for 90 min at 20 °C, centrifuged, and the phases separated and analyzed. Apart from the HNO_3_ concentration, all conditions were kept constant throughout the experiment.

#### Influence of the SO_3_-Ph-BTBP concentration

The influence of the SO_3_-Ph-BTBP concentration was evaluated by contacting an aqueous phase consisting of 0.5 mol L^−1^ HNO_3_, 3 mol L^−1^ NH_4_NO_3_ and 5, 10, 25, 50, 100 or 150 mmol L^−1^ of SO_3_-Ph-BTBP with an organic phase consisting of bulk loaded 0.2 mol L^−1^ TODGA in Aliquat-336 nitrate. The samples were shaken for 90 min at 20 °C, centrifuged and the phases separated. Apart from the ligand concentration, all conditions were kept constant throughout the series.

#### Extraction from HAR with aliphatic AmSel

Prior to extraction, the masking agent cyclohexanediaminetetraacetic acid (CDTA) was added to the HAR to obtain a 0.05 mol L^−1^ solution. This prevents zirconium and palladium from being coextracted in the first step.^48^ As the CDTA degrades over time, affecting the efficiency of the masking, the solution was always freshly prepared. To demonstrate the original AmSel system, 1 mL of simulated HAR in 4 mol L^−1^ HNO_3_ solution with 0.05 mol L^−1^ CDTA spiked with radiotracers was contacted with 1 mL of 0.2 mol L^−1^ TODGA in 5 vol% 1-octanol in *n*-dodecane. The sample was mechanically shaken for 1 h at 20 °C and was afterwards centrifuged and the phases were separated. Of the loaded organic phase, 0.5 mL was contacted with 0.5 mL of stripping solution. Two sets of stripping solutions were created: the first set consisted of 20 mmol L^−1^ of SO_3_-Ph-BTBP in 0.3, 0.5, 0.7 or 0.9 mol L^−1^ of HNO_3_ solution, and the second set consisted of 3, 5, 7 or 9 mmol L^−1^ of SO_3_-Ph-BTBP in 0.3 mol L^−1^ of HNO_3_ solution, conditions already tested by Wagner *et al.*^38^ The samples were shaken for 1 h at 20 °C, centrifuged, and the phases were separated and analyzed with alpha and gamma spectrometry.

To analyze the behavior of the fission products, an analogous extraction was performed without added radiotracer. 1 mL of simulated HAR solution in 4 mol L^−1^ HNO_3_ with 0.05 mol L^−1^ CDTA was contacted with 1 mL of 0.2 mol L^−1^ TODGA in 5 vol% 1-octanol in *n*-dodecane. The sample was mechanically shaken at 20 °C for 1 h, after which the sample was centrifuged, and the phases separated. The depleted aqueous phase was then analyzed with ICP-MS, and the extraction efficiencies were calculated and can be found in Table 1b. Of the loaded organic phase, 0.5 mL was contacted with 0.5 mL of stripping solution consisting of 4 mmol L^−1^ of SO_3_-Ph-BTBP in 0.3 mol L^−1^ HNO_3_ solution. This sample was again shaken for 1 h at 20 °C and afterwards centrifuged and the phases separated. The aqueous phase was then also analyzed with ICP-MS, and stripping efficiencies were calculated and can be found in Table 1c.

#### Extraction from HAR with ionic liquid AmSel

Analogously to the aliphatic AmSel system, extraction and stripping experiments with the ionic liquid AmSel system were performed on a simulated HAR solution. During extraction, an identical aqueous phase consisting of HAR solution in 4 mol L^−1^ HNO_3_ with 0.05 mol L^−1^ CDTA was used, and contacted with an organic phase consisting of 0.2 mol L^−1^ TODGA in Aliquat-336 nitrate. This was shaken for 1 h at 20 °C, after which the samples were centrifuged, and the phases separated and analyzed with alpha and gamma spectrometry. Afterwards, 0.5 mL of loaded organic phase was contacted with 0.5 mL of stripping solution consisting of 50 mmol L^−1^ of SO_3_-Ph-BTBP and 3 mol L^−1^ NH_4_NO_3_ in 0.5 mol L^−1^ HNO_3_. The samples were shaken for 90 min at 10, 20, 30 or 40 °C, centrifuged, and the phases were separated and analyzed.

To analyze the behavior of the fission products, an analogous extraction was performed without the addition of radiotracer. 1 mL of simulated HAR in 4 mol L^−1^ HNO_3_ solution with 0.05 mol L^−1^ CDTA was contacted with 1 mL of 0.2 mol L^−1^ TODGA in Aliquat-336 nitrate. The sample was shaken for 1 h at 20 °C, after which the sample was centrifuged, and the phases separated. The depleted aqueous phase was then analyzed with ICP-MS, and the extraction efficiencies were calculated and can be found in Table 1d. 0.5 mL of the loaded organic phase was then contacted with 0.5 mL of stripping solution consisting of 50 mmol L^−1^ of SO_3_-Ph-BTBP and 3 mol L^−1^ of NH_4_NO_3_ in 0.5 mol L^−1^ of HNO_3_ solution. The sample was then shaken for 90 min at 40 °C. After shaking the sample was centrifuged and the phases separated. Based on the starting composition of the HAR and the concentrations of the fission products in the aqueous phases after loading and stripping, stripping efficiencies were calculated and can be found in Table 1e.

### Spectroscopic methods

#### Inductively coupled plasma mass spectrometry

Inactive aqueous samples were analyzed using ICP-MS, on a ThermoFisher Scientific X2 series II ICP-MS instrument. Samples were prepared by adding 100 μL of the aqueous phase to 9.9 mL of a 2% HNO_3_ solution. Pd could not be accurately measured during the HAR experiments due to interference of Y, Mo, and Zr. The uncertainty on all of the measurements is 10%. Due to the LoD of the measurements, distribution ratios could only be calculated up to the following values: Sm: 3000, Eu: 2200, Gd: 2000, Dy: 930, Yb: 800, Y: 1200. For La, Ce, Pr, and Nd all measurements were above the LoD.

#### Gamma spectrometry

Both aqueous and organic radioactive samples were analyzed through gamma spectrometry. Weighed aliquots of 300 μL were pipetted into 1.5 mL vials and analyzed. The samples were measured on a Canberra High Purity Germanium detector (model: GC2520) with a DSA-1000 Multi-Channel Analyzer and analyzed with Genie2000 software. The samples were measured until relevant peaks (at 59.51 keV for ^241^Am and at 121.8 keV for ^152^Eu) showed at least 10 000 counts to keep the relative counting uncertainty of the corrected net peak area below 1%.^49^ Very low activity samples were measured overnight.

#### Alpha spectrometry

Thin layer alpha samples were prepared for both organic and aqueous phases. These were prepared on 20 mm diameter C-1S cupped stainless steel planchets acquired from GA-MA and Associates, Inc., Florida, USA. Both organic and aqueous phases were diluted 1 : 10 with the corresponding solvent prior to sample preparation to limit interference of the dissolved ligands and salts. Of the aqueous samples *ca.* 10 μL was pipetted into the middle of the planchet and the exact mass of the sample was determined by weighing on an analytical balance. One drop of both a 1 mol L^−1^ HNO_3_ solution and a 25% NH_4_OH solution were then added to obtain a smoother distribution. The planchets were then dried under an infrared lamp and subsequently burned with a gas torch to fix the sample. For organic samples 5 μL of sample was pipetted onto a planchet and the exact mass of the sample was determined by weighing on an analytical balance. These were then also dried under the infrared lamp and afterwards burned with a gas torch. The samples were measured by alpha spectrometry using a Canberra Alpha Analyst spectrometer, equipped with Passivated Implanted Planar Silicon (PIPS) alpha detectors, and analyzed with Canberra Apex Alpha software. The alpha spectra were analyzed by measuring the area of the ^241^Am peak at 5.5 MeV and the ^244^Cm peak at 5.8 MeV. Both aqueous and organic phases showed baseline separation, with little overlap of the Am and Cm peaks.

#### UV-VIS spectrometry

UV-VIS spectrometry was used to observe the behavior of SO_3_-Ph-BTBP during stripping. Samples were measured in HELLMA precision SUPRASIL^®^ quartz cells, with a path length of 10 mm, with a Shimadzu UV-1800 spectrophotometer. The obtained data were analyzed with Shimadzu UVProbe software. Two calibration curves were constructed according to the Lambert–Beer law by measuring solutions with 0.1, 1, 2.5, 5 and 10 mmol L^−1^ of SO_3_-Ph-BTBP in 0.3 mol L^−1^ HNO_3_ at 488 and 652 nm. Aqueous samples were diluted 1 : 3 with a solution of the same HNO_3_ concentration as the sample and were measured together with a blank containing a solution with the same concentration of HNO_3_ but without any ligand. Absorbances were measured at both wavelengths 488 nm and 652 nm and fitted to the calibration curves to obtain concentrations.

### Mathematical calculations

Distribution ratios *D* were calculated at equilibrium (except in kinetics experiments) as the ratio of the concentration of an element in the organic phase over the activity or concentration in the aqueous phase at equilibrium. This formula is represented in eqn (1).1
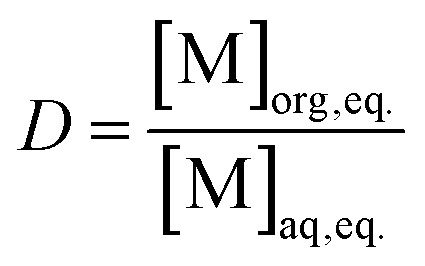


The concentrations of the elements in the organic phases were calculated based on ICP-MS measurements of the feed solution ([M]_aq feed, eq._), depleted aqueous phases after loading ([M]_aq depl, eq._), and aqueous phases after stripping ([M]_aq strip, eq._). When the aqueous and organic phases have equal volumes, eqn (2) describes the *D* value of the stripping.2
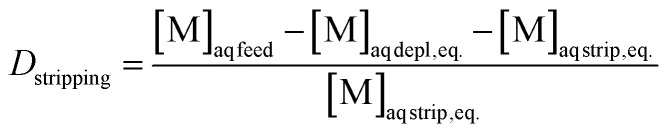


The separation factor of two elements was obtained by calculating the ratio between their distribution ratios, with the higher value as the numerator to obtain a value larger than 1. The separation factor was calculated by using eqn (3). Alpha spectrometry data were always used for the calculation of the SF_Cm/Am_.3
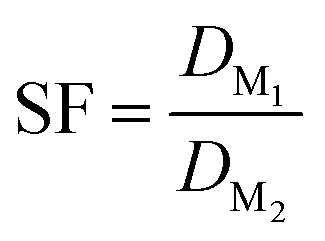


Extraction efficiencies were determined by dividing the calculated metal concentration in the organic phase after extraction by the metal concentration in the aqueous phase before extraction. Stripping efficiencies were determined by dividing the metal concentration in the aqueous phase after stripping by the calculated metal concentration in the organic phase before stripping. The formulas are represented in eqn (4) and (5) respectively.4
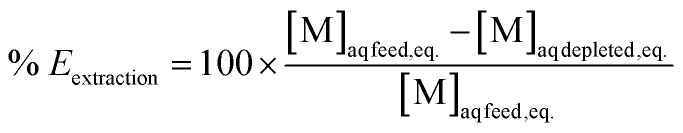
5
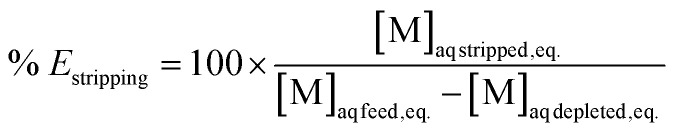


## Results and discussion

### SO_3_-Ph-BTBP behavior during extraction

During initial tests it was immediately observed that when stripping with SO_3_-Ph-BTBP in solutions containing less than 3 mol L^−1^ of HNO_3_ was performed, the BTBP was partially extracted by the Aliquat-336 nitrate organic phase. This was observed both visually, as the usually bright yellow aqueous phase became pale and the colorless organic phase became yellow, and quantitatively, as distribution ratios deviated from the expected trend at low concentrations of nitric acid. This extraction is undesirable as it greatly decreases the efficiency of the stripping. Besides the effect on the distribution ratios during stripping, the presence of SO_3_-Ph-BTBP in the organic phase also complicates its recycling, and as it introduces sulfur into the organic waste stream it also causes problems at the end-of-life during waste treatment.

**Fig. 2:**
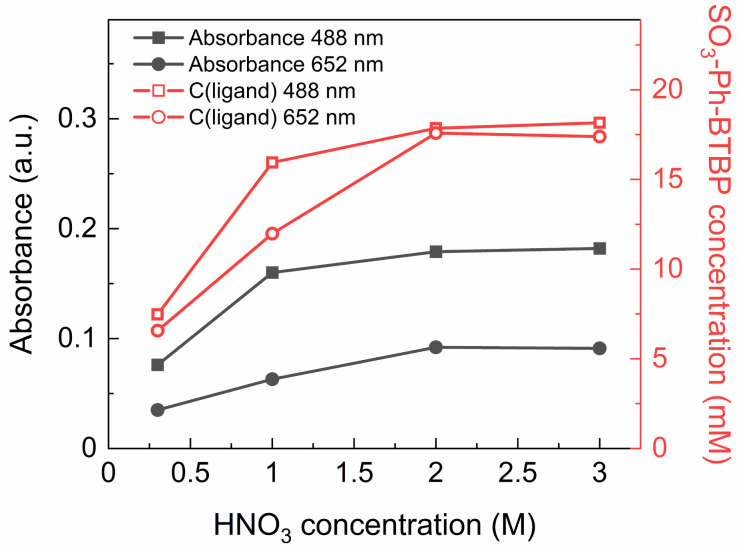
Absorbance at 488 and 652 nm in function of HNO_3_ concentration (black lines, closed symbols) and calculated SO_3_-Ph-BTBP concentration at 488 and 652 nm in aqueous phase in function of HNO_3_ concentration (red lines, open symbols) for extraction with TODGA in Aliquat-336. Organic phase consisting of 0.05 mol L^−1^ TODGA in Aliquat-336 nitrate loaded with Ln(iii) by extraction from 3 mol L^−1^ HNO_3_ solution. Initial aqueous phase consisting of 20 mmol L^−1^ SO_3_-Ph-BTBP in HNO_3_ solution with varying HNO_3_ concentrations. Shaking time = 1 h, A/O = 1, *T* = 20 °C.

In order to better understand the degree to which SO_3_-Ph-BTBP is extracted during stripping, a loaded organic phase consisting of 0.05 mol L^−1^ TODGA in Aliquat-336 nitrate was stripped with aqueous phases containing 20 mmol L^−1^ SO_3_-Ph-BTBP in various HNO_3_ concentrations between 0.3 and 3 mol L^−1^. Afterwards, the aqueous phases were analyzed with UV-VIS spectrometry. The samples were diluted 1 : 3 with a HNO_3_ solution of the same concentration. Blanks were prepared with a corresponding HNO_3_ concentration but without any SO_3_-Ph-BTBP. The absorbance was measured at 488 and 652 nm (see Fig. 2 – black closed symbols), and by fitting these on the calibration curves the concentration of SO_3_-Ph-BTBP was calculated (see Fig. 2 – red open symbols).

It was found that after stripping with a 0.3 mol L^−1^ HNO_3_ solution, only 7.5 mmol L^−1^ of the initial 20 mmol L^−1^ SO_3_-Ph-BTBP remained in the aqueous phase. The major part was extracted into the organic phase. When stripping was attempted in a 3 mol L^−1^ HNO_3_ solution, 18 mmol L^−1^ of SO_3_-Ph-BTBP remained in the aqueous phase. A possible explanation for this occurrence is an ion exchange mechanism of the SO_3_-Ph-BTBP with the nitrate from the Aliquat-336 nitrate as represented in eqn (6).^50^ At low HNO_3_ concentrations SO_3_-Ph-BTBP is not fully protonated, bearing an overall negative charge which lets it enter an ion exchange reaction with the nitrates from the organic phase.^51^ As the HNO_3_ concentration increases, the BTBP becomes protonated at the N-sites causing the ligand's negative charge to diminish.^52^ This decrease in negative charge removes the driving force of the exchange reaction, preventing further extraction of the ligand into the organic phase. The p*K*_a_ value of SO_3_-Ph-BTBP has been determined through UV/VIS spectrometry in a previous study and was found to be 2.2, indicating that a significant portion of the complexant would be protonated at the used HNO_3_ concentrations.^37^ High HNO_3_ concentrations prevent the loss of the SO_3_-Ph-BTBP ligand to the organic phase but this also causes a negative effect on the stripping efficiency. This leads to distribution ratios for Am(iii) far above 1 as increasing the HNO_3_ concentration leads to stronger extraction by TODGA and to SO_3_-Ph-BTBP being less available for complexation with the An(iii).^37,53^ An alternative strategy is therefore necessary to prevent extraction of the ligand to the organic phase, particularly at low acidities.

6 SO_3_-Ph-BTBP_aq_^4−^ + 4[A336]_org_^+^ + 4[NO_3_]_org_^−^ ⇆ 4[NO_3_]_aq_^−^ + 4[A336]_org_^+^ + SO_3_-Ph-BTBP_org_^4−^

### Influence of the NH_4_NO_3_ concentration on the extraction of SO_3_-Ph-BTBP

**Fig. 3:**
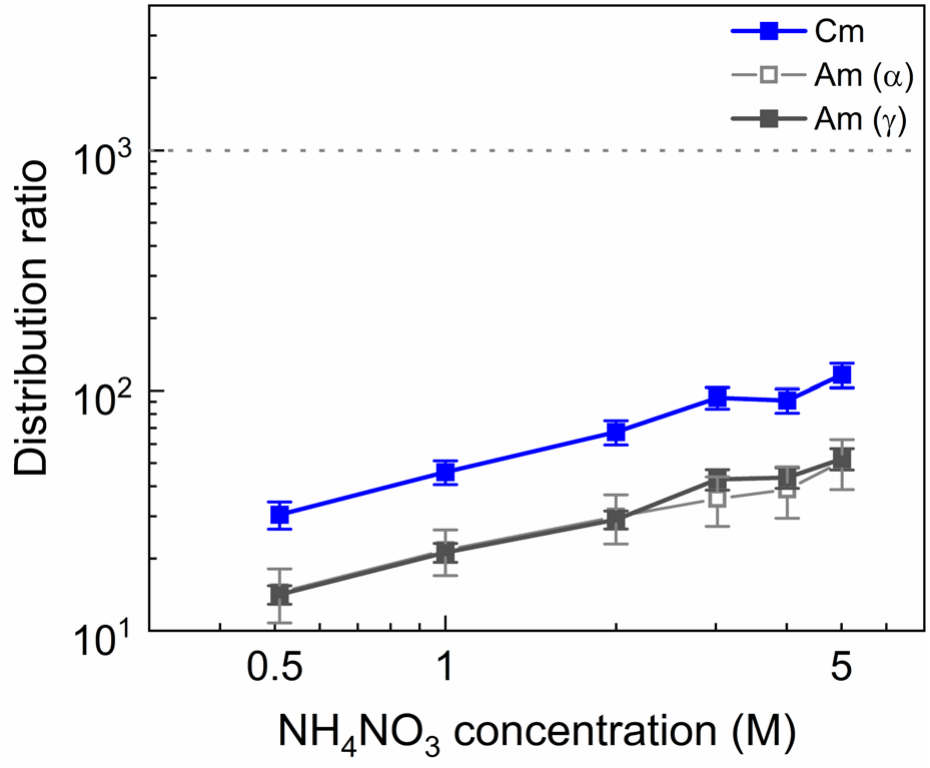
Distribution ratios of Am(iii) and Cm(iii) for stripping with SO_3_-Ph-BTBP as a function of NH_4_NO_3_ concentration. Organic phase consisting of 0.2 mol L^−1^ TODGA in Aliquat-336 nitrate loaded with ^241^Am(iii), ^244^Cm(iii), ^152^Eu(iii) and inactive Ln(iii) by extraction from 3 mol L^−1^ HNO_3_ solution. Aqueous phases consisting of NH_4_NO_3_ and 20 mmol L^−1^ SO_3_-Ph-BTBP in 0.5 mol L^−1^ HNO_3_ solution. Shaking time = 90 min, A/O = 1, *T* = 20 °C.

It was explained previously how using higher HNO_3_ concentrations is an effective method to counteract the ion exchange mechanism that leads to the extraction of SO_3_-Ph-BTBP to the organic phase. However, this leads to elevated distribution ratios for Am due to a combination of increased TODGA extraction and decreased availability of SO_3_-Ph-BTBP as a result of protonation. A possible alternative is raising the aqueous nitrate concentration with a nitrate salt in order to prevent the ion exchange of NO_3_^−^ ions through the principle of Le Châtelier without significantly altering the pH. To test this, NH_4_NO_3_ was chosen as it is compliant with the CHON principle, minimizing issues during end-of-life waste treatment. Bulk loaded organic phase was stripped containing between 0.5 and 5 mol L^−1^ NH_4_NO_3_ and 20 mmol L^−1^ SO_3_-Ph-BTBP in 0.5 mol L^−1^ HNO_3_. The results are presented in Fig. 3.

An increase of the distribution ratios was observed for both Am and Cm when the NH_4_NO_3_ concentration was increased. This is explained by the extraction mechanism of TODGA, that requires NO_3_^−^ ions to form a neutral complex with M^3+^ ions in order for extraction to take place.^54,55^ This salting-out effect has also already been observed with NaNO_3_ as a source of NO_3_^−^ ions in previous studies where extractions with TODGA dissolved in Aliquat-336 nitrate were investigated. ^152^Eu aqueous activities fell below the LoD of gamma spectrometry, and distribution ratios of Eu would therefore be well in excess of 1000.

The addition of NH_4_NO_3_ to the stripping solution appears to have a positive effect on the ligand behavior. In experiments where 1 mol L^−1^ or more of NH_4_NO_3_ was added to the aqueous phase, no discoloration of the organic phase could be observed by the naked eye. The exact ligand concentration remaining in the aqueous phase could however not be determined by means of UV-VIS spectrophotometry due to interference from NH_4_NO_3_. This retention of the ligand could be explained by the saturation of the aqueous phase with NO_3_^−^ ions from the dissolution of NH_4_NO_3_. This prevents the nitrate ions in Aliquat-336 nitrate from entering an ion exchange reaction with the deprotonated SO_3_-Ph-BTBP through the principle of Le Châtelier. In order to ensure that no extraction of SO_3_-Ph-BTBP occurs even when higher ligand concentrations are used, 3 mol L^−1^ of NH_4_NO_3_ was chosen for all subsequent stripping experiments.

### Stripping kinetics

**Fig. 4:**
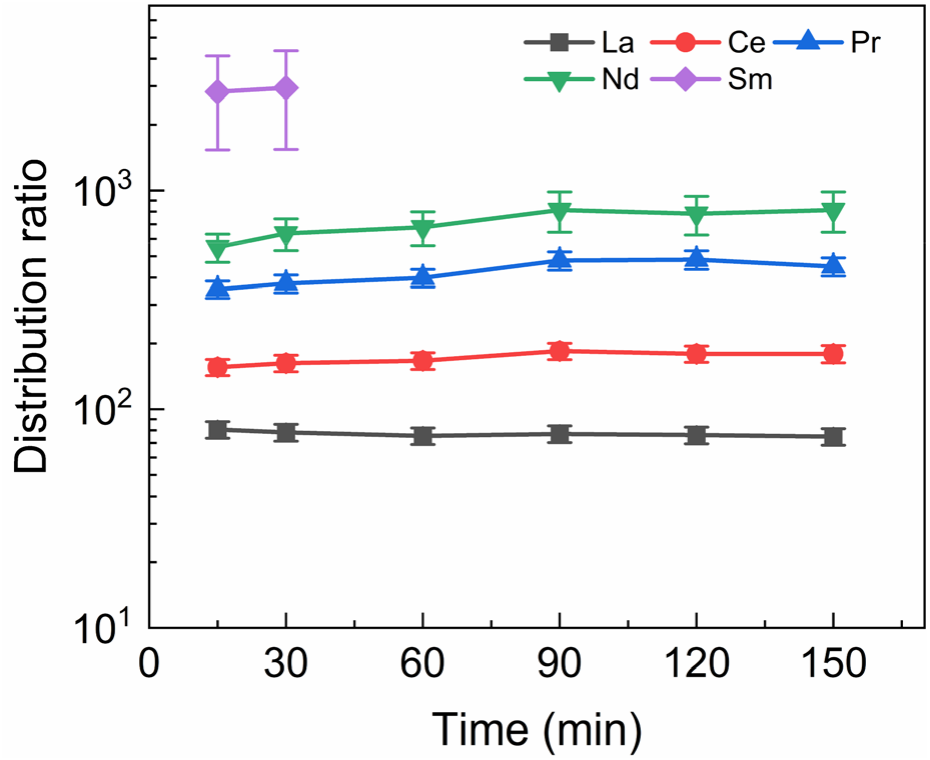
Distribution ratios of Ln(iii) for stripping with SO_3_-Ph-BTBP as a function of time. Organic phase consisting of 0.2 mol L^−1^ TODGA in Aliquat-336 nitrate loaded with inactive Ln(iii) by extraction from 3 mol L^−1^ HNO_3_ solution. Aqueous phases consisting of 20 mmol L^−1^ SO_3_-Ph-BTBP and 3 mol L^−1^ NH_4_NO_3_ in 0.5 mol L^−1^ HNO_3_ solution. A/O = 1, *T* = 20 °C.

**Fig. 5:**
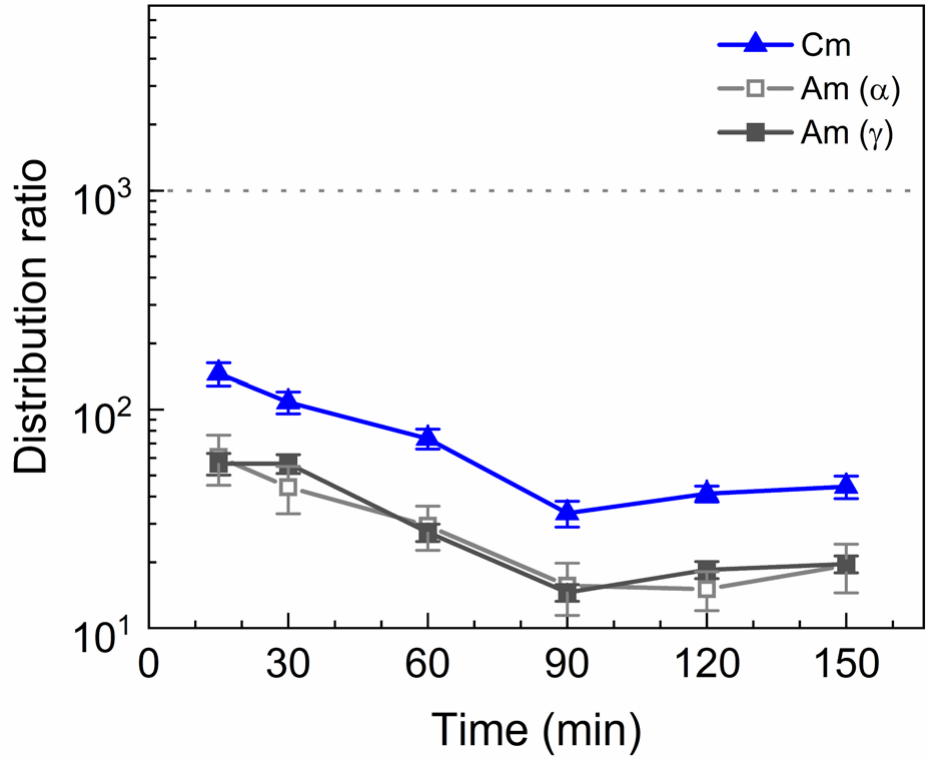
Distribution ratios of Am(iii) and Cm(iii) for stripping with SO_3_-Ph-BTBP as a function of time. Organic phase consisting of 0.2 mol L^−1^ TODGA in Aliquat-336 nitrate loaded with ^241^Am(iii), ^244^Cm(iii), ^152^Eu(iii) and inactive Ln(iii) by extraction from 3 mol L^−1^ HNO_3_ solution. Aqueous phases consisting of 20 mmol L^−1^ SO_3_-Ph-BTBP and 3 mol L^−1^ NH_4_NO_3_ in 0.5 mol L^−1^ HNO_3_ solution. A/O = 1, *T* = 20 °C.

A kinetics study was performed of the stripping of Am, Cm and the Ln from a loaded organic phase consisting of 0.2 mol L^−1^ TODGA in Aliquat-336 nitrate. The samples were shaken between 15 and 150 min. The results of the inactive experiment are presented in Fig. 4 and the results of the active experiment are presented in Fig. 5.

Fig. 4 shows fast kinetics for the measurable lanthanides, although a slight increase in distribution ratios can be observed for several of the lanthanides. La and Ce are in equilibrium already after 15 min of shaking, while Pr and Nd show an increase in distribution ratios until around 90 min of shaking. For Sm distribution ratios could only be calculated for 15 and 30 min of shaking, as longer shaking times resulted in aqueous concentrations under the LoD of ICP-MS. The lanthanides Eu, Gd, and Dy, as well as Y, are only minimally stripped and their aqueous concentration falls below the LoD for all samples.

Americium and curium show notably slower kinetics, only achieving equilibrium after 90 min of shaking. The ^152^Eu radiotracer activity could not be measured by gamma spectrometry because it was below the LoD. This is in contrast to the kinetics observed by Wagner *et al.* in the aliphatic AmSel system, where reportedly equilibrium during stripping with SO_3_-Ph-BTBP is already achieved in less than 7 min.^38^ The longer shaking times required to reach equilibrium are a result of the higher viscosity of Aliquat-336 nitrate in comparison to dodecane. Slower kinetics have already previously been observed for ionic liquid-based solvents. In a study investigating the extraction of Am(iii) and Ln(iii) with a diluent consisting of 0.05 mol L^−1^ TODGA in Aliquat-336 nitrate equilibrium was achieved after 30 min of shaking, whereas when aliphatic solvents are used only a few min of shaking are usually required.^45^ A similar increase in shaking time was also observed for a system containing *N*,*N*,*N*′,*N*′-tetraethylhexyldiglycolamide (TEHDGA) in the [N_1888_][NO_3_] ionic liquid.^56^ In light of these findings, samples were shaken for 90 min to reach equilibrium in all subsequent experiments.

### Influence of the HNO_3_ concentration on the stripping

**Fig. 6:**
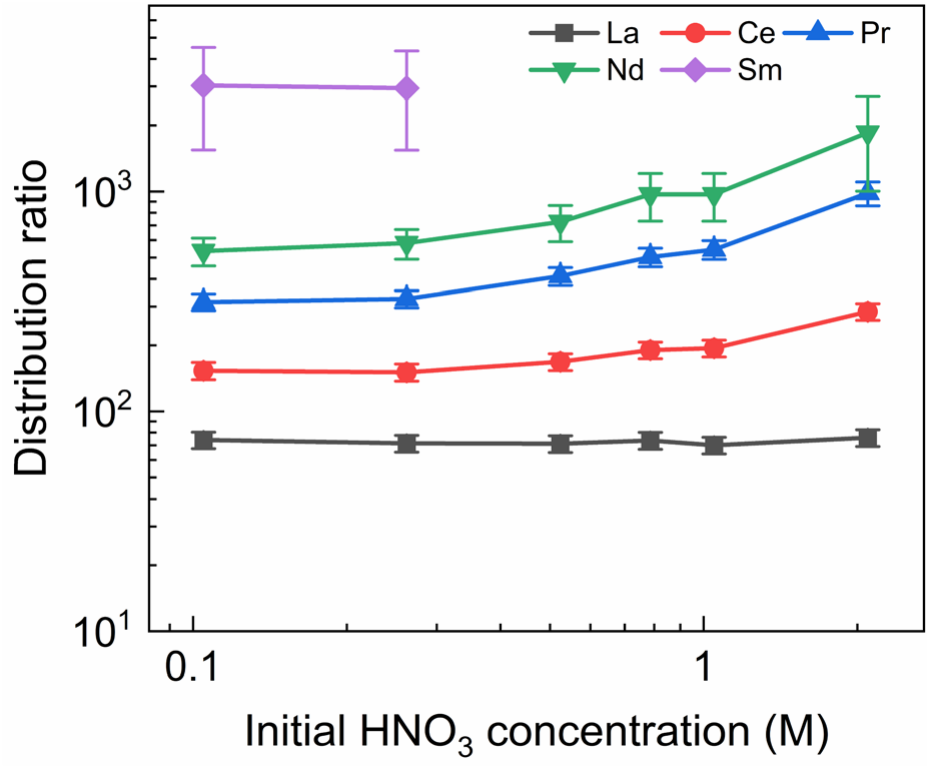
Distribution ratios of Ln(iii) for stripping with SO_3_-Ph-BTBP as a function of initial HNO_3_ concentration. Organic phase consisting of 0.2 mol L^−1^ TODGA in Aliquat-336 nitrate loaded with Ln(iii) by extraction from 3 mol L^−1^ HNO_3_ solution. Aqueous phases consisting of 20 mmol L^−1^ SO_3_-Ph-BTBP and 3 mol L^−1^ NH_4_NO_3_ in HNO_3_ solution. Shaking time = 90 min, A/O = 1, *T* = 20 °C.

**Fig. 7:**
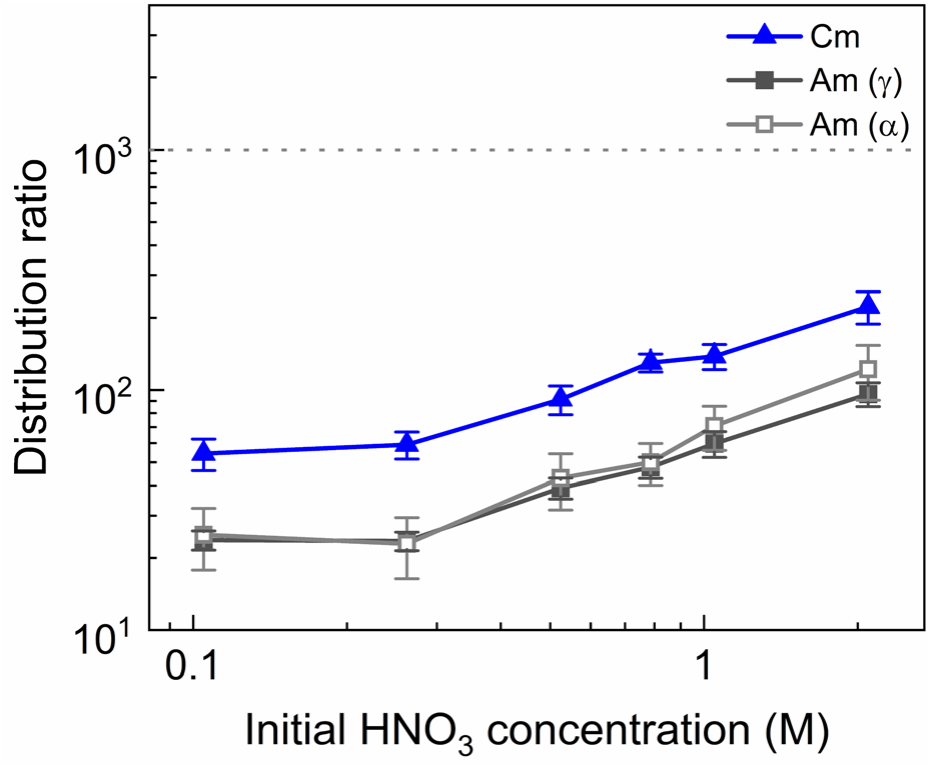
Distribution ratios of Am(iii) and Cm(iii) for stripping with SO_3_-Ph-BTBP as a function of initial HNO_3_ concentration. Organic phase consisting of 0.2 mol L^−1^ TODGA in Aliquat-336 nitrate loaded with ^241^Am(iii), ^244^Cm(iii), ^152^Eu(iii) and inactive Ln(iii) by extraction from 3 mol L^−1^ HNO_3_ solution. Aqueous phases consisting of 20 mmol L^−1^ SO_3_-Ph-BTBP and 3 mol L^−1^ NH_4_NO_3_ in HNO_3_ solution. Shaking time = 90 min, A/O = 1, *T* = 20 °C.

To investigate the influence of the HNO_3_ concentration on the stripping in the Aliquat-336 nitrate based system, a series of experiments was performed with nitric acid concentrations in the stripping solutions varying between 0.1 mol L^−1^ and 2 mol L^−1^, and fixed concentrations of 20 mmol L^−1^ for SO_3_-Ph-BTBP and 3 mol L^−1^ for NH_4_NO_3_. Stripping was performed from a bulk loaded organic phase consisting of 0.2 mol L^−1^ TODGA in Aliquat-336 nitrate. The results of the inactive experiment are presented in Fig. 6 and the results of the radioactive experiment are presented in Fig. 7.

No color change of the organic phase was observed during the experiment, indicating that addition of NH_4_NO_3_ is able to prevent extraction of the SO_3_-Ph-BTBP ligand to the organic phase. A comparison of Fig. 7 and 3 also shows that an increase in the NH_4_NO_3_ concentration does not raise the distribution ratios of Am and Cm as much as an increase in the HNO_3_ concentration does. At a starting NO_3_^−^ concentration of 3.5 mol L^−1^, slightly lower distribution ratios are observed for an aqueous phase containing 3 mol L^−1^ NH_4_NO_3_ + 0.5 mol L^−1^ HNO_3_ than are observed for an aqueous phase containing 0.5 mol L^−1^ NH_4_NO_3_ + 3 mol L^−1^ HNO_3_. For a starting NO_3_^−^ concentration of 5 mol L^−1^, the system containing 3 mol L^−1^ NH_4_NO_3_ + 2 mol L^−1^ HNO_3_ shows distribution ratios that are double the ratios obtained for a system containing 4.5 mol L^−1^ NH_4_NO_3_ + 0.5 mol L^−1^ HNO_3_. This difference is also observed in the slopes of the two curves: whereas varying the NH_4_NO_3_ concentration yields a slope of around 0.57, varying the HNO_3_ concentration gives a slope closer to 0.67. This shows that, especially for higher nitrate concentrations, it is preferable to increase the NH_4_NO_3_ concentration rather than the HNO_3_ concentration as to limit the increase in distribution ratios for the actinides. Further optimization of these concentrations would however be required to find the ideal concentrations of the various components in the stripping solution.

In general, the lanthanides show higher distribution ratios when stripping is performed at higher HNO_3_ concentrations. This increase appears to be stronger for heavier than for lighter lanthanides, with distribution ratios for La showing almost no change between 0.1 mol L^−1^ and 2 mol L^−1^ of HNO_3_ while for Nd the ratios more than triples over this range. This difference in influence of the acid concentration on the distribution of various lanthanides was also observed, to a lesser extent, by Wagner *et al.* in the AmSel system, and is likely a result of the hard O-donors of TODGA showing preference for the heavier lanthanides.^38,57^ This also explains the observed trend between the lanthanides whereby progressively higher distribution ratios are observed with increasing atomic number. The combination of a hard donor organic extractant and a soft donor aqueous extractant leads to a separation of the metal ions based on their hardness according to Pearson's HSAB principle. Due to the lanthanide contraction, the hardness of the lanthanides increases with their atomic number, leading to stronger extraction by TODGA and weaker stripping by SO_3_-Ph-BTBP.

As in the kinetics experiment, Eu, Gd, Dy, and Y are not stripped sufficiently to obtain an aqueous concentration above the LoD of ICP-MS, and distribution ratios could therefore not be determined. For Sm distribution ratios could only be calculated when stripping was performed with 0.10 and 0.25 mol L^−1^ of HNO_3_. It must be noted that these experiments show distribution ratios for all of the Ln that were orders of magnitude higher than what was observed in the aliphatic AmSel system.^38^ This can at least partially be explained by the difference in methodologies between this and the original AmSel study. Whereas the original study added radiotracers and lanthanides to the stripping solution and performed extractions with a bare organic phase, this study performed an initial extraction to load the organic phase with the desired lanthanides and actinides. This initial extraction step was performed with a 3 mol L^−1^ HNO_3_ solution and was preceded by two pre-equilibration steps in order to saturate the organic phase with HNO_3_. The TODGA-[A336][NO_3_] system has previously been shown to extract a significant concentration of HNO_3_ during extractions, and this HNO_3_ is carried over to the stripping step.^45^ This results in significantly lower equilibrium concentrations of HNO_3_ in Wagner's study, and higher equilibrium concentrations in this study, changing the extraction by TODGA. However as HNO_3_ extraction during the loading step is unavoidable in a scaled up, continuous process, it can significantly alter the efficiency of the subsequent stripping step. Therefore it was chosen to perform the loading step in this study for a more realistic evaluation of the extraction system. This can explain the differences in distribution ratios that were observed since it is well-known that the *D* values of the extraction of trivalent Ln and An by TODGA increase with increasing nitric acid concentration.^54^ Furthermore, the influence of the Aliquat-336 nitrate should not be underestimated, as it inherently contains high NO_3_^−^ concentrations and also contributes to the extraction of metal ions.^45,58^

A larger variation in distribution ratios is observed for the actinides in Fig. 7, with distribution ratios for Am and Cm showing a roughly fourfold increase from 0.1 mol L^−1^ to 2 mol L^−1^ of HNO_3_. As in the previous two experiments, the Eu distribution ratios were so high that the activity of ^152^Eu in the aqueous phase could not be determined. At no point does the distribution ratio of Am fall below 1, indicating that 20 mmol L^−1^ of SO_3_-Ph-BTBP is not sufficient to achieve stripping. Similarly to what was observed in the aliphatic AmSel system, a drop in separation factor between Cm and Am is observed at higher nitric acid concentrations. For HNO_3_ concentrations below 1 mol L^−1^ the SF_Cm/Am_ varies between 2.2 and 2.5, for 1 mol L^−1^ it drops to 1.9, and for 2 mol L^−1^ the SF_Cm/Am_ further drops to 1.8. This was explained in previous studies by the protonation of the coordinating nitrogen atoms of the SO_3_-Ph-BTBP ligand at higher nitric acid concentrations.^38^ Separation between Am and the Ln decreases with increasing HNO_3_ concentrations, as Am shows a stronger increase in distribution ratios than the Ln. At the lowest tested HNO_3_ concentration a SF_La/Am_ value of 3.1 is obtained, slightly higher than the SF_Cm/Am_ value of 2.2. When 2 mol L^−1^ of HNO_3_ is used, no separation between La and Am is observed anymore. Since the distribution ratio for Am does not drop below 1 for any of the tested conditions, a follow up experiment was performed in which the ligand concentration was increased in order to facilitate Am stripping.

### Influence of the SO_3_-Ph-BTBP concentration on the stripping

**Fig. 8:**
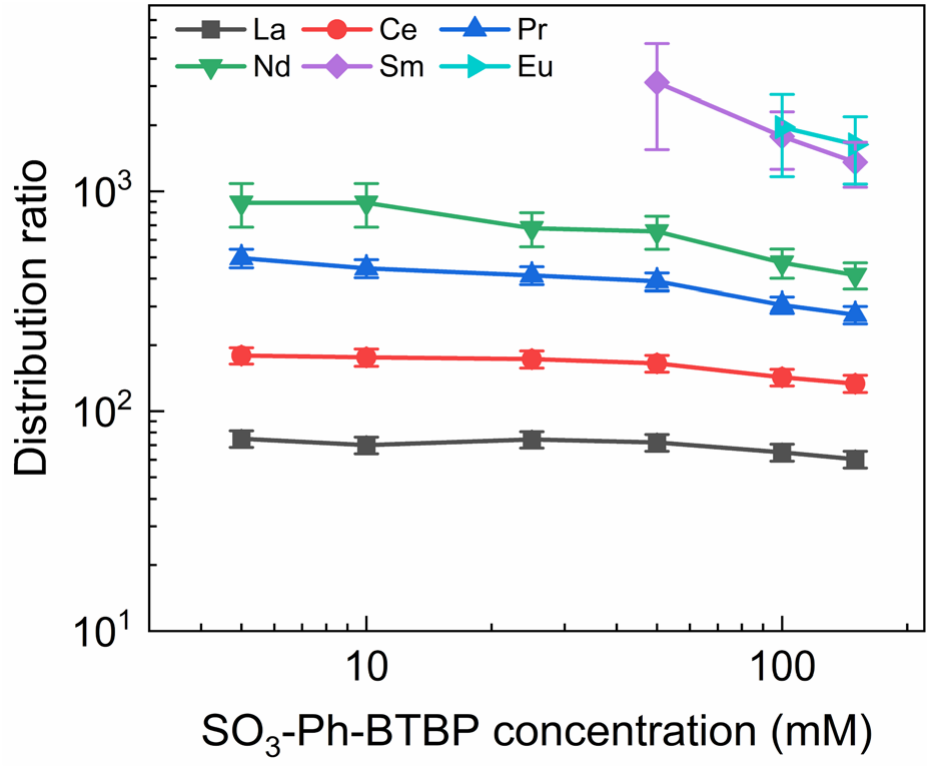
Distribution ratios of Ln(iii) for stripping with SO_3_-Ph-BTBP as a function of SO_3_-Ph-BTBP concentration. Organic phase consisting of 0.2 mol L^−1^ TODGA in Aliquat-336 nitrate loaded with Ln(iii) by extraction from 3 mol L^−1^ HNO_3_ solution. Aqueous phases consisting of SO_3_-Ph-BTBP and 3 mol L^−1^ NH_4_NO_3_ in 0.5 mol L^−1^ HNO_3_ solution. Shaking time = 90 min, A/O = 1, *T* = 20 °C.

**Fig. 9:**
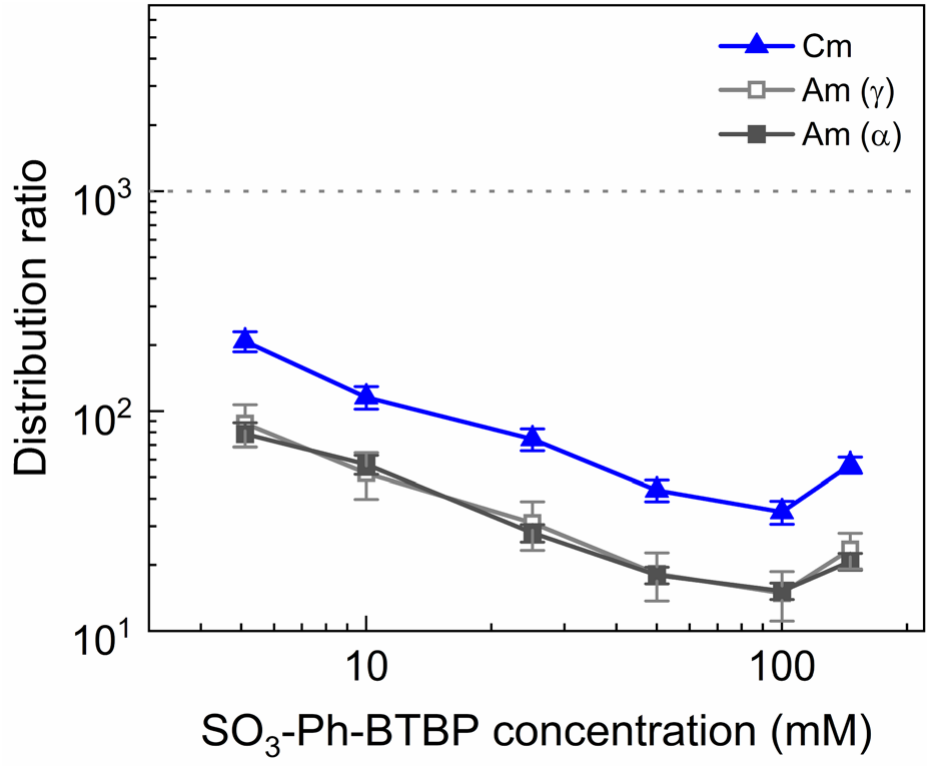
Distribution ratios of Am(iii) and Cm(iii) for stripping with SO_3_-Ph-BTBP as a function of SO_3_-Ph-BTBP concentration. Organic phase consisting of 0.2 mol L^−1^ TODGA in Aliquat-336 nitrate loaded with ^241^Am(iii), ^244^Cm(iii), ^152^Eu(iii) and inactive Ln(iii) by extraction from 3 mol L^−1^ HNO_3_ solution. Aqueous phases consisting of SO_3_-Ph-BTBP and 3 mol L^−1^ NH_4_NO_3_ in 0.5 mol L^−1^ HNO_3_ solution. Shaking time = 90 min, A/O = 1, *T* = 20 °C.

To investigate the influence of the SO_3_-Ph-BTBP concentration on the stripping in the Aliquat-336 nitrate system, a series of samples was stripped at a constant HNO_3_ concentration of 0.5 mol L^−1^ and a constant NH_4_NO_3_ concentration of 3 mol L^−1^, with SO_3_-Ph-BTBP concentrations between 5 and 150 mmol L^−1^. Stripping was performed from a loaded organic phase consisting of 0.2 mol L^−1^ TODGA in Aliquat-336 nitrate. The results of the inactive experiment are presented in Fig. 8 and the results of the active experiment are presented in Fig. 9.

Even at 150 mmol L^−1^ of ligand, the highest tested concentrations, no color change of the organic phase was observed, indicating that NH_4_NO_3_ prevents the extraction of the ligand. The light lanthanides appear largely unaffected by the increase in ligand concentration. La and Ce show only a slight decline in distribution ratios throughout the series and Pr and Nd only show a significant drop in distribution ratios when more than 50 mmol L^−1^ of ligand is used for stripping. Although Sm and Eu show steeper slopes at high ligand concentrations, the high uncertainty of these distribution ratios makes it difficult to draw any conclusions. Gd, Dy, Yb, and Y once again show minimal stripping leading to aqueous concentrations below the LoD of ICP-MS. Similarly, Sm and Eu also show too high distribution ratios at low ligand concentrations to measure, and can only be measured from respectively 50 mmol L^−1^ and 100 mmol L^−1^ of SO_3_-Ph-BTBP in the stripping solution.

The distribution ratios for the actinides show a more pronounced decrease with increasing ligand concentrations. As was observed in previous tests, 20 mmol L^−1^ of SO_3_-Ph-BTBP is not sufficient to achieve stripping of Am. Raising the ligand concentration has a decreasing effect on the distribution ratios of Am and Cm, reaching a minimum of 15 and 38 for these respectively when 100 mmol L^−1^ of ligand is used. When an even higher concentration is used, the distribution ratios of both Am and Cm show a strong increase. This might indicate that there is precipitation of the formed complexes, although no precipitation was visually observed and no such behavior was observed for the lanthanides. This point was therefore left out during the following calculations of the slope.

Plotting log *D*_Am(iii),α_*versus* log[SO_3_-Ph-BTBP] gives a slope of −0.62 (*R*^2^ = 0.989), and a similar slope is obtained for the results obtained by gamma spectrometry. When log *D*_Cm(iii),α_ is plotted as a function of log[SO_3_-Ph-BTBP], a slope of −0.61 (*R*^2^ = 0.986) is obtained. These values deviate from the −1.3 slope found by Wagner *et al.*,^38^ but also imply the formation of 1 : 1 complexes, despite Time-Resolved Laser-induced Fluorescence Spectroscopy (TRLFS) experiments proving the formation of 1 : 2 complexes. This discrepancy was already observed in previous slope analyses of the SO_3_-Ph-BTBP ligand, as well as for SO_3_-Ph-BTP, but is usually not observed for other BTBP or BTP type ligands.^37,38,59^ Distribution ratios for Am and Cm are again higher than those found by Wagner *et al.*,^38^ and can again be explained by differences the extraction system as well as differences in methodology. As was also observed in previous experiments in this paper, Eu could again not be measured in the aqueous phase by gamma spectrometry because its activity concentration was below the LoD. Separation factors SF_Cm/Am_ varied between 2.2 and 2.4 throughout the series, and did not seem to be significantly influenced by the ligand concentration. This behavior corresponds well with what was observed in the aliphatic AmSel system, although slightly higher separation factors in the 2.5–3.0 range were found there.^38^ The separation factors between the lanthanides and Am show in general an increasing trend. Although no separation is observed between La and Am at the lowest concentration of SO_3_-Ph-BTBP, the SF_La/Am_ reaches a maximum value of 4.3 when 100 mmol L^−1^ of ligand is used. The same lanthanide trend can be observed as with previous experiments, showing higher distribution ratios with increasing atomic number. Although the distribution ratios for Sm and Eu are outside of the range of detection for most points, at 100 and 150 mmol L^−1^*D* values could be calculated and were found to be similar. This was also observed in the original AmSel study.^38^

### Demonstration on a simulated highly active raffinate

**Fig. 10:**
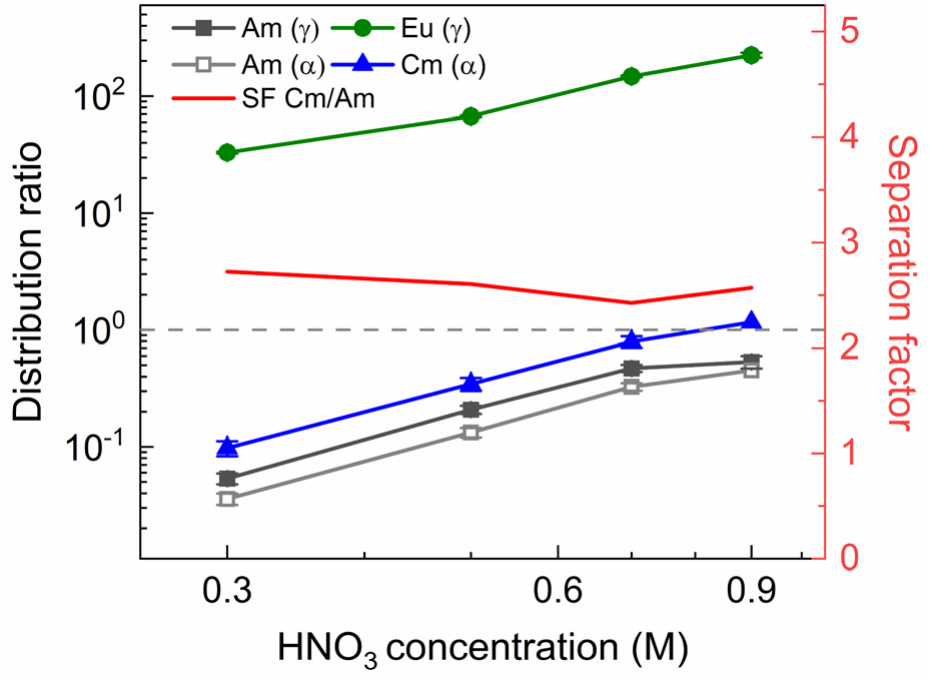
Distribution ratios of Am(iii), Cm(iii), and Eu(iii) for stripping with SO_3_-Ph-BTBP an organic phase loaded with a simulated HAR as a function of HNO_3_ concentration. Organic phase consisting of 0.2 mol L^−1^ TODGA in 5 vol% 1-octanol in *n*-dodecane loaded with a simulated HAR solution + radiotracer in 4 mol L^−1^ HNO_3_. Aqueous phases consisting of 20 mmol L^−1^ SO_3_-Ph-BTBP in HNO_3_ solution. Shaking time = 60 min, A/O = 1, *T* = 20 °C.

**Fig. 11:**
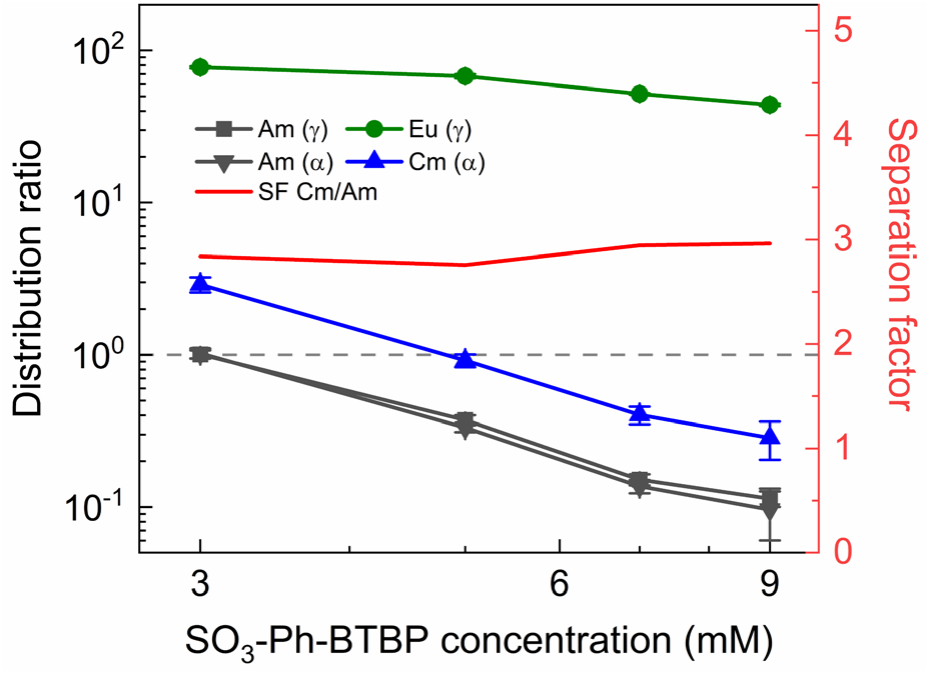
Distribution ratios of Am(iii), Cm(iii), and Eu(iii) for stripping with SO_3_-Ph-BTBP, from an organic phase loaded with a simulated HAR, as a function of SO_3_-Ph-BTBP concentration. Organic phase consisting of 0.2 mol L^−1^ TODGA in 5 vol% 1-octanol in *n*-dodecane loaded with a simulated HAR solution + radiotracer in 4 mol L^−1^ HNO_3_. Aqueous phases consisting of SO_3_-Ph-BTBP in 0.3 mol L^−1^ HNO_3_ solution. Shaking time = 60 min, A/O = 1, *T* = 20 °C.

To determine the viability of both the conventional as well as the ionic liquid AmSel system, both systems were tested on a simulated PUREX highly active raffinate. So far, no such tests with a simulated HAR have been reported for the AmSel system. The exact composition of this simulated HAR can be found in Table 1a. Extraction from this simulated HAR was performed with an organic phase consisting of 0.2 mol L^−1^ TODGA in *n*-dodecane with 5 vol% 1-octanol, the same concentrations as used by Wagner *et al.*^38^ This loaded organic phase was subsequently stripped with either solutions containing a fixed ligand concentration (20 mmol L^−1^) and varying HNO_3_ concentrations (0.3–0.9 mol L^−1^ HNO_3_), or solutions containing a fixed HNO_3_ concentration (0.3 mol L^−1^) and varying ligand concentrations (3–9 mmol L^−1^ SO_3_-Ph-BTBP). These conditions were chosen to allow for comparison with the results obtained by Wagner *et al.*^38^ The results of the experiment in which the HNO_3_ concentration is varied are presented in Fig. 10 and the results of the experiment in which the ligand concentration is varied are presented in Fig. 11.

The observed trends corroborate with the results of the AmSel optimization study published by Wagner *et al.*,^38^ with increasing *D* values at higher concentrations of HNO_3_, and decreasing *D* values at higher ligand concentrations. Furthermore, good separation between on one hand Eu and Am, and on the other hand Cm and Am is observed. In the HNO_3_ dependence experiments a Cm(iii)/Am(iii) separation factor of around 2.8 ± 0.1 was calculated, and a factor of around 2.6 ± 0.1 was obtained for the ligand dependence experiment. SF_Eu/Am_ showed significant variation, reaching values between 77 ± 5 and 390 ± 50 in [SO_3_-Ph-BTBP] the variation experiment, and between 620 ± 70 and 310 ± 20 in the [HNO_3_] variation experiment. When log *D*_Am(iii),α_ and log *D*_Cm(iii),α_ are plotted *versus* log[SO_3_-Ph-BTBP], a slope of −2.16 (*R*^2^ = 0.9935) and −2.21 (*R*^2^ = 0.9934) is obtained, respectively. When the same is done for log *D*_Eu(iii),γ_ a slope of −0.52 is obtained (*R*^2^ = 0.9441). It is remarkable that the slopes obtained for Am and Cm in this experiment imply the formation of 1 : 2 complexes, which is different from what was observed in our previous experiments. Furthermore, the formation of a 1 : 2 complex is in agreement with TRLFS data.^37,38^ Eu on the other hand shows a similar slope to what was found for the actinides in previous experiments, which corresponds rather to 1 : 1 complexes, and does not agree with TRLFS data.^37^ The cause of the variation between the Am and Cm slopes and the Eu slope is unknown. In the [HNO_3_] variation experiment a favorable region for selective Am stripping, where Am shows distribution ratios below 1 and Cm shows distribution ratios above 1, was found at 0.9 mol L^−1^ of HNO_3_ at a ligand concentration in the aqueous phase of 20 mmol L^−1^. In the [SO_3_-Ph-BTBP] variation experiment, where the starting HNO_3_ concentration is kept at 0.3 mol L^−1^, this region is located between 3 and 5 mmol L^−1^ of SO_3_-Ph-BTBP. It is within these regions that selective extraction can take place in a continuous setup, with the latter being preferred as it requires less SO_3_-Ph-BTBP and less HNO_3_ to achieve the separation, while still having enough ligand available to strip Am which occurs in PUREX HARs in concentrations around 0.4 mmol L^−1^.^60^ After identification of the separation region, an inactive experiment was performed to analyze the distribution of the fission products. This experiment was performed analogously to the active experiment, with as stripping conditions 0.3 mol L^−1^ HNO_3_ and 4 mmol L^−1^ SO_3_-Ph-BTBP, the concentrations for which *D*(Am) < 1 and *D*(Cm) > 1. Based on the starting composition of the HAR and the concentrations of the fission products in the aqueous phases after loading and stripping, both extraction and stripping efficiencies were calculated and are shown respectively in Table 1b and in Table 1c. Stripping efficiencies were not calculated for elements of which the extraction efficiency in the loading was less than 1%. For these elements the starting concentrations during the stripping step were too low to calculate accurate stripping efficiencies. These elements would also not be present in the organic phase during the stripping step in a multi-stage continuous setup. During stripping, only molybdenum and strontium are stripped to a significant extent along with Am(iii) and Cm(iii). For molybdenum this is not a problem, as molybdenum is largely retained in the aqueous phase during the extraction step. However, strontium is extracted by TODGA, and its tendency to be co-stripped with Am will require additional scrubbing step or masking agents to prevent this from happening.^19^ Other elements that are also stripped to a small extent are ruthenium, zirconium and the light lanthanides (La, Ce, Pr and Nd), but as the distribution ratios remain above one, their separation from americium remains feasible. A SF_La/Am_ of 5.1 is found, and the SF_Eu/Am_ is around 100. With the exception of strontium, americium was therefore successfully separated from the remaining fission products.

**Fig. 12:**
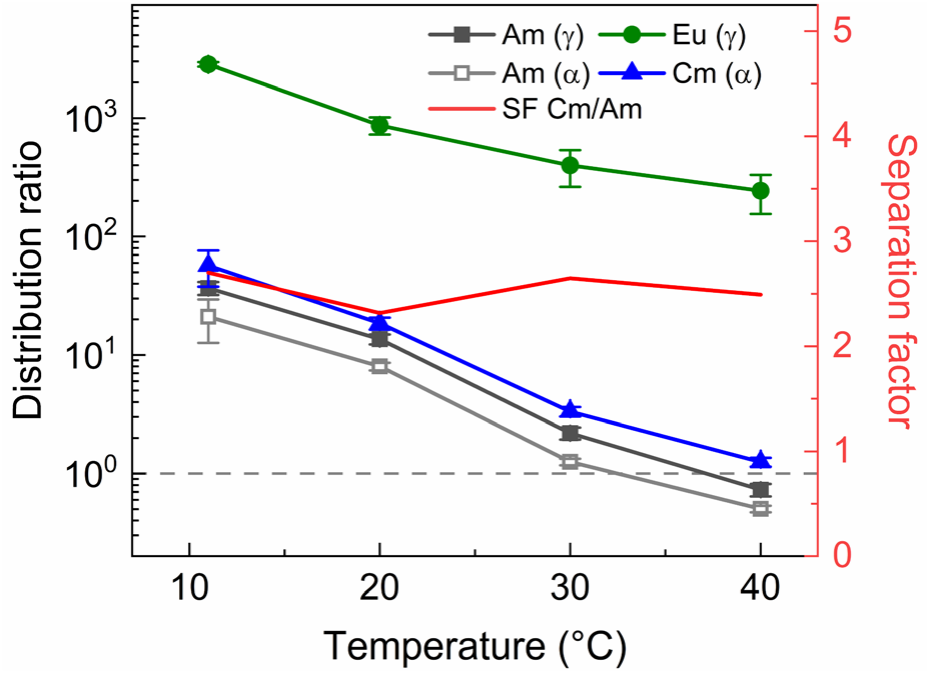
Distribution ratios of Am(iii), and Cm(iii), and Eu(iii) for stripping with SO_3_-Ph-BTBP, from an organic phase loaded with a simulated HAR, as a function of temperature. Organic phase consisting of 0.2 mol L^−1^ TODGA in Aliquat-336 nitrate loaded with a simulated HAR solution + radiotracer in 4 mol L^−1^ HNO_3_. Aqueous phases consisting of 50 mmol L^−1^ SO_3_-Ph-BTBP and 3 mol L^−1^ NH_4_NO_3_ in 0.5 mol L^−1^ HNO_3_ solution. Shaking time = 90 min, A/O = 1.

Next to the original AmSel process, the ionic liquid based AmSel process was demonstrated with a simulated HAR. An organic phase with 0.2 mol L^−1^ of TODGA was used for the HAR experiments with the Aliquat-336 nitrate based AmSel process as was used in the previous screening experiments. However with this solvent, no stripping conditions were found that yielded distribution ratios for Am lower than 1. In order to achieve this, a third parameter was varied during stripping experiments: the temperature. TODGA extraction is known to be an exothermic reaction, therefore, lower *D* values are obtained at higher temperatures.^61^ An additional benefit is that higher temperatures were also shown to improve extraction kinetics for extractions of Am(iii), Cm(iii), and Eu(iii) with Aliquat-336 nitrate.^46^ 50 mmol L^−1^ of SO_3_-Ph-BTBP in 0.5 mol L^−1^ HNO_3_ was chosen as the stripping solution. This ligand concentration was chosen to ensure no precipitation occurs during stripping, as was the case in Fig. 9, even when performed with the significantly higher metal concentrations expected of a simulated HAR. 3 mol L^−1^ NH_4_NO_3_ was added to prevent loss of the ligand to the organic phase. The samples were shaken at various temperatures for 90 min, centrifuged, and the phases separated. The results are presented in Fig. 12.

Raising the temperature indeed lowers the distribution ratios, and at 40 °C Am/Cm separation is possible. At this temperature a SF_Cm/Am_ of 2.5 ± 0.2 and a SF_Eu/Am_ of 330 ± 30 were observed. An inactive experiment was subsequently performed analogously to the active experiment in order to evaluate the behavior of the fission products within the ionic liquid based variant of the AmSel process. The stripping solution consisted of 0.5 mol L^−1^ HNO_3_, 3 mol L^−1^ NH_4_NO_3_, and 50 mmol L^−1^ SO_3_-Ph-BTBP, and stripping was performed at 40 °C. Based on the starting composition of the HAR and the concentrations of the fission products in the aqueous phases after loading and stripping, both extraction and stripping efficiencies were calculated and are shown respectively in Table 1d and in Table 1e. Stripping efficiencies were again not calculated for elements of which the extraction efficiency in the loading step was less than 1%.

During loading most fission products are retained in the aqueous phase, with only the lanthanides and yttrium being significantly extracted to the organic phase. Zinc, ruthenium and zirconium are extracted notably more in the ionic liquid system when compared to the original AmSel system. Molybdenum and strontium were extracted to a lesser extent in the ionic liquid system. In the ionic liquid system, strontium was largely retained in the aqueous phase during loading, as was shown in previous studies.^45^ This is an improvement over the original AmSel system where strontium is coextracted during loading. As with the aliphatic system, most of the molybdenum and strontium in the organic phase is stripped by SO_3_-Ph-BTBP in the ionic liquid system. Overall, a significant improvement is observed in separation between Am(iii) and the fission products. During loading, the fission products other than the lanthanides were generally extracted slightly more when compared to the original AmSel system, except for molybdenum and strontium. These fission products are however retained far more in the Aliquat-336 nitrate during the stripping step. Especially the light lanthanides (La, Ce, Pr and Nd) show significantly improved separation from the actinides when compared to the original AmSel system. A SF_La/Am_ of 35 was found, and the SF_Eu/Am_ exceeds 300. This is a sevenfold increase in separation factor between lanthanum and americium, and threefold increase for europium, compared to the aliphatic AmSel system. These separation factors can also be compared to a similar system that employs a hydrophobic complexant (CyMe_4_BTPhen) and hydrophilic DGA (TEDGA) in Aliquat-336 nitrate. There significantly higher separation factors were obtained, with SF_Am/Cm_ = 3.1–3.9, SF_Am/La_ > 75, and SF_Am/Eu_ ≥ 3000.^46^ However the behavior of fission products was not tested, and based on literature several fission products (Cu, Pd, Cd, Ag, Sn, Ni, and Mo) are expected to be co-extracted if no additional masking agents are used.^62^ Nevertheless, this experiment shows that, the AmSel system in Aliquat-336 nitrate has similar separation factors between Cm and Am as the original AmSel system, and in addition it provides better separation between Am and the fission products, especially the light lanthanides and strontium, and thus a more pure Am stream could be obtained.

## Conclusions

In this study an ionic liquid based variant of the AmSel system was developed by introducing Aliquat-336 nitrate as an alternative organic diluent. This system was optimized using batch extractions, followed by a demonstration on a simulated highly active raffinate. As the original AmSel system had not yet been tested on a simulated HAR, its performance was also evaluated for comparison. The use of Aliquat-336 nitrate instead of an aliphatic diluent shows improved separation between Am and the fission products, particularly the lighter lanthanides and strontium, when tested on a simulated PUREX raffinate. Am/Cm separation was maintained, showing separation factors around 2.5, similar to the original AmSel system with an aliphatic organic phase, leading overall to a final Am stream of higher final purity.

The ionic liquid based variant shows slower extraction kinetics due to its inherent higher viscosity, although this can be mitigated by working at higher temperatures.^46^ In the case shown in this paper, higher temperatures of 40 °C are required to enable a practical Am/Cm separation (distribution ratio of Cm above 1 and of Am below 1 at the specific conditions – see Fig. 12).

The system has shown a tendency to extract the water-soluble ligand into the organic phase, decreasing extraction efficiency and complicating waste treatment. It was possible to mitigate this extraction through the addition of NH_4_NO_3_ to the stripping solution. However, salting-out agents could better be avoided, as these have a significant contribution to final waste volumes.^63^ Further speciation studies on the ligand behavior during the extraction could provide more insight on how to prevent loss of the ligand to the organic phase.

Another aspect of the AmSel system to consider is that the SO_3_-Ph-BTBP ligand is not compliant with the CHON principle and complicates waste treatment regardless of which solvents are used. Nevertheless, AmSel remains interesting due to its ability to selectively separate Am in highly acidic conditions, and its ability to separate Am from both the lanthanides and Cm at the same time. Further research, perhaps with new task-specific ionic liquids and/or variations of the SO_3_-Ph-BTBP complexant, could further improve the extraction system.

## Author contributions

Filip Kolesar: solvent extraction experiments, sample preparation, radioanalytical measurements, data treatment, conceptualization, investigation, writing – original draft. Karen Van Hecke: supervision, writing – review & editing. Péter Zsabka: HAR preparation, supervision, writing – review & editing. Ken Verguts: supervision, writing – review & editing. Koen Binnemans: conceptualization, supervision, writing – review & editing. Thomas Cardinaels: conceptualization, funding acquisition, supervision, writing – review & editing.

## Conflicts of interest

There are no conflicts of interest to declare.

## Supplementary Material
